# Effectiveness and Compatibility of a Novel Sustainable Method for Stone Consolidation Based on Di-Ammonium Phosphate and Calcium-Based Nanomaterials

**DOI:** 10.3390/ma12183025

**Published:** 2019-09-18

**Authors:** Cecilia Pesce, Ligia M. Moretto, Emilio F. Orsega, Giovanni L. Pesce, Marco Corradi, Johannes Weber

**Affiliations:** 1 Department of Architecture and Built Environment, Faculty of Engineering and Environment, Northumbria University Newcastle, Newcastle upon Tyne NE1 8ST, UK; giovanni.pesce@northumbria.ac.uk; 2 Department of Molecular Sciences and Nanosystems, Ca’ Foscari University of Venice, 30172 Mestre Venice, Italy; moretto@unive.it (L.M.M.); ors-ef@unive.it (E.F.O.); 3 Department of Engineering, University of Perugia, 06125 Perugia, Italy; marco.corradi@unipg.it; 4 Institute of Art and Technology, Conservation Sciences, University of Applied Arts Vienna, 1010 Vienna, Austria; johannes.weber@uni-ak.ac.at

**Keywords:** consolidation, nanomaterials, calcium carbonate, hydroxyapatite, limestone, sandstone, cultural heritage, scanning electron microscopy

## Abstract

External surfaces of stones used in historic buildings often carry high artistic value and need to be preserved from the damages of time, especially from the detrimental effects of the weathering. This study aimed to test the effectiveness and compatibility of some new environmentally-friendly materials for stone consolidation, as the use thereof has been so far poorly investigated. The treatments were based on combinations of an aqueous solution of di-ammonium phosphate (DAP) and two calcium-based nanomaterials, namely a commercial nanosuspension of Ca(OH)_2_ and a novel nanosuspension of calcite. The treatments were applied to samples of two porous stones: a limestone and a sandstone. The effectiveness of the treatments was assessed using scanning electron microscopy coupled with energy-dispersive X-ray spectroscopy, Fourier-transform infrared spectroscopy, ultrasound pulse velocity test, colour measurements, and capillary water absorption test. The results suggest that the combined use of DAP and Ca-based nanosuspensions can be advantageous over other commonly used consolidants in terms of retreatability and physical-chemical compatibility with the stone. Some limitations are also highlighted, such as the uneven distribution and low penetration of the consolidants.

## 1. Introduction

Stone is one of the most durable construction materials used in historic buildings. However, over time, the stone can be subject to various degradation processes leading to physical and chemical modifications [1,2,3,4,5]. Although these effects may be limited to the surface and negligible to the structural stability of the affected buildings, they can represent a major problem in carved decorative elements of artistic value, where any detail should be preserved.

The challenge for conservators and material scientists involved in stone conservation has always been to find a way to stop or delay the effects of these degradation processes. Since the 19th century, craftsmen and restorers have been applying a variety of products to stone elements with the aim of regaining strength and cohesion. However, in many cases, these attempts relied on empirical evidence and were based on a hit-or-miss approach and on the use of locally available consolidants [6]. A more scientifically-sound approach gradually followed over the last century, leading to the development of novel products specially tailored for stone consolidation. Although some good results have been obtained for indoor stone surfaces, the development of long-term solutions for stone exposed to outdoor weathering is still an open challenge, in particular on account of climatic changes effects [7].

Deteriorated sedimentary stones typically show loss of intergranular cohesion due to the impoverishment of the natural interstitial binder. This usually affects an external stone layer and is the result of chemical processes or physical and thermal stresses that cause an abrupt variation of the chemical, physical and/or mechanical properties within the stone [5,7,8]. To re-establish cohesion within the altered layer and continuity between this and the underlying sound material, a consolidation treatment is usually necessary. The products used in such interventions are liquids (solutions or colloidal suspensions) able to penetrate the stone pore network where a solid phase with binding properties is precipitated. Ideally, consolidants should be able to significantly improve some measurable properties of the stones (i.e., the abrasion resistance), have good adhesion with the substrate, be compatible with the substrate and allow future treatments [6,7,8,9,10]. Currently, the most common products used for stone consolidation are lime-based, polymeric, and silicate-based consolidants [6,7].

Lime-based treatments, such as limewater and lime milk, have been traditionally used for the consolidation of calcareous substrates (e.g., limestone), and are considered highly compatible because of their chemical and structural affinity with the substrate, made up of calcium carbonate [11]. However, it is generally acknowledged that they have very limited penetration and little consolidation effect [6,12,13]. Furthermore, being water-based, their application may entail the mobilisation of salts already within the substrate that, upon re-crystallisation, can have detrimental effects on the treated stone [13].

Polymeric consolidants, such as acrylic solutions (e.g., Paraloid^®^ B72) and emulsions (e.g., Primal^®^ AC 33) have been extensively used for stone consolidation [14]. These products can strengthen the substrate when they solidify upon solvent evaporation and are appreciated for their good adhesive properties [10]. However, many questions have been recently raised about their long-term stability, mostly affected by photo-oxidative degradation [15,16,17,18]. Furthermore, some of these products are reported to have low impregnation capacity and to form hard crusts, leading to severe detachment when used in highly porous stones and in combination with silicate-based consolidants [6].

Alkoxysilanes, primarily methyltrimethoxysilane (MTMOS) and tetraethoxysilane (TEOS), have been the most widely used consolidants over the past twenty years. They impart strength to the stone by forming a silica gel inside the pore network upon polymerisation of the silanol units. Their popularity is due to several advantageous properties, such as good penetration, thermal and oxidative stability, long-term experience, as well as their commercial availability [6,9]. The main issue related to their use is the high sensitivity to environmental conditions: relative humidity and pH variations, and presence of soluble salts or clay minerals can affect the curing process, leading to surface whitening and micro-fissures formation [6,17]. Furthermore, silicate consolidants are often stated to hardly bond to calcareous substrates [6,19,20] and are subject to shrinking upon cross-linking.

Over the last years, various innovative stone consolidants have been developed and tested with particular attention on their compatibility and environmental impact [16]. 

The use of nanoparticles for the conservation of various artistic materials (e.g., frescoes and paper) has been promising [16,21,22,23]. Several advantages were identified for the use of calcium hydroxide nanoparticles over the traditional limewater treatment, such as a higher calcium concentration (up to 50 g/L, against 1.7 g/L of a saturated solution of Ca(OH)_2_), and the use of an organic carrier allowing for less pronounced agglomeration, easier capillary absorption, quick evaporation and reduced diffusion of soluble salts [11,24]. Treatments based on alcoholic dispersions of calcium hydroxide nanoparticles have been successfully applied for the conservation of historical renders, porous limestone, marble and wall paintings [25,26,27,28,29,30].

A further innovative approach is the use of ammonium phosphates solutions, introduced by some recent pioneering studies for consolidation of carbonate stones and for some types of calcium-bearing silicate stones, with several advantages such as high compatibility with carbonate stones and the possibility to use concentrated solutions [10,31,32]. Ammonium phosphates are a family of salts extensively used in several industrial fields [33,34]. Among them, di-ammonium phosphate (DAP, formula (NH_4_)_2_HPO_4_) is the reagent most commonly used in stone consolidation. When in contact with a calcareous substrate, DAP reacts with the calcium ions in solution according to Reaction (1):

5 Ca^2+^ + 3 PO_4_^3−^ + OH^−^ → Ca_5_(PO_4_)_3_(OH). (1)

The reaction product is hydroxyapatite (HAP), a mineral widely studied in biology and medicine [35]. HAP shows advantageous properties as a consolidant, such as its high stability and mineralogical compatibility with calcite [10,36]. Alongside HAP, other minerals form as a result of Reaction (1), i.e., metastable Ca-P phases and precursors, as well as amorphous calcium phosphate, depending on the surrounding conditions. Although the relationship between the chemical-mineralogical nature of such Ca-P phases and their respective consolidation action is not fully understood yet, most of them are less soluble than calcite and, therefore, are likely to contribute to the overall consolidation effect [32]. 

Some recent studies have been carried out to investigate the effectiveness and suitability of DAP treatments on weathered stones. Results thus far are promising and include a significant increase in dynamic elastic modulus and tensile strength, unaffected pore size distribution and moisture transfer properties, re-treatability of the consolidated surfaces, as well as full effectiveness after 48 h from the treatment [10,20,31,37,38,39].

This study aimed to evaluate the effectiveness and compatibility of the combinations of an aqueous solution of DAP and two Ca-based nanomaterials, namely an ethanol-based nanosuspension of Ca(OH)_2_ and a novel aqueous nanosuspension of calcite. The advantages brought by the use of these products are manifold: (i) the low environmental impact of nanosuspensions and phosphate solutions with respect to other common formulations based on solvents harmful to the health and environment ([16] and references therein]); (ii) calcium-based nanomaterials are aimed at boosting HAP formation and allow DAP application to calcium-poor substrates, as it had been previously tested using limewater poultice as additional calcium source [37]; (iii) the reduced particle size should promote penetration and inter-molecular interaction with the substrate [40]; and (iv) the high concentration allows for reducing the number of necessary applications, and therefore introducing a lessened amount of water into the stone, unlike limewater treatment.

## 2. Materials and Methods

### 2.1. Tested Lithotypes

Two porous substrates were used to evaluate the effectiveness of the consolidation treatments: a carbonate and a silicate-based stone. Both stones were used in the past as a building material and, due to their homogeneity and the range of well-defined pore sizes, can be considered as reference testing substrates [19,41]. Both substrates were among those selected in the NanoCathedral European project (GA 646178, Call NMP-21-2014: Materials-based solutions for protection or preservation of European cultural heritage) [42], within which this research was conducted.

The silicate stone was a quartzitic arenite called Schlaitdorfer sandstone, quarried in Baden-Württemberg (Germany). This stone has been used as a construction material since the 19th century for structural and decorative use in monuments and graves. Some of the most famous examples of use are the Cathedrals of Cologne, Münster, and Ulm [41,43]. The stone is a white-yellow coarse-grained clastic arenite. Clasts mainly consist of coarse fragments of rocks (about 1 mm), accumulated in layers and orientated parallel to the bedding planes. Quartz is the dominant phase (72%) and is distributed in tight concretions; several inclusions of feldspar and micas can be found, along with strongly-corroded microcline orthoclase, carbonate, and marl. The clasts are cemented by an interstitial matrix (14%) of fine-grained dolomite, quartz, and clay minerals (mainly non-swelling kaolinite and illite). Typical deterioration phenomena affecting Schlaitdorfer sandstone are sanding, flaking, scaling and crust formation [8,41,43].

Before testing, the slabs of sound stone used in this research were subject to artificial ageing by thermal treatment, carried out at the Institute of Geotechnics, Research Centre of Engineering Geology, Vienna University of Technology according to the method developed by Ban et al. [44]. The treatment allowed for the widening of cracks and detachment of crystals in a homogeneous fashion through the profile specimen, mimicking the properties of naturally weathered material. Upon thermal treatment, the open porosity increased from 15% to 18%, accompanied by a widening of the average pore diameter from 0.3 µm to 0.6 µm [45].

**Figure 1:**
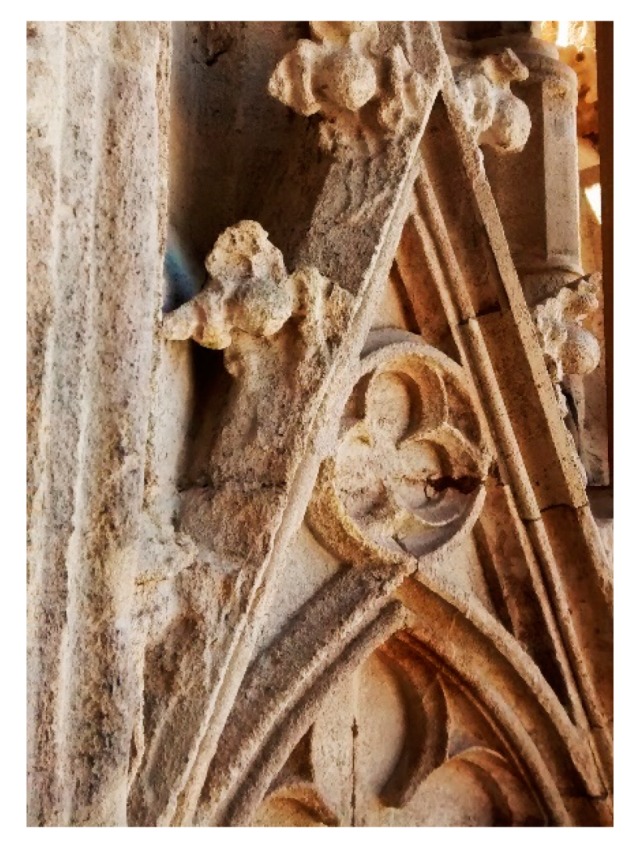
An outdoor decorated frame of Auer limestone of St. Stephen’s Cathedral façade in Vienna (Austria), showing extensive erosion and loss of detail after exposure to weathering agents.

The carbonate stone used was a biogenic calcareous arenite called Auer limestone [46,47,48]. This stone was quarried in Au am Leithagebirge (Austria) and has been extensively used as building and ornamental material since the 14th century for its aesthetical qualities and large availability. It was used for the construction of many important buildings in Vienna, such as St. Stephen’s Cathedral and Minoriten Church. Petrographic analyses revealed that Auer limestone mainly consists of skeletal fragments grains (coralline red algae, foraminifera, and bivalves) and other carbonate clasts, partially orientated parallel to the bedding planes [46]. Calcite is the dominant phase, whereas terrigenous siliceous inclusions are mainly quartz, muscovite and plagioclase. Clasts are cemented by fine-grained dogtooth calcite. The stone can be described as a light yellow-brown, coarse-grained (up to 2 mm grain size) detritic sedimentary rock with total open porosity about 40% and pores ranging from a few micrometres to millimetre size. This stone is subject to gypsum formation as surface crust by sulphation of its grain cement and to granular disintegration by exposure to freeze–thaw and salt crystallisation cycles (Figure 1) [49]. Because of its remarkably high porosity, sound limestone was used for the specimens. Unlike the sound sandstone, the number of active pores in the unaged limestone allowed for effective absorption of the consolidants, so that no artificial ageing was required.

Specimens of selected geometry and dimensions were produced by wet-cutting the stone slabs with a table saw. The cut specimens were brushed and water-rinsed to remove dust, and subsequently dried in an oven at 100 °C until constant weight. Prisms of 50 mm × 20 mm × 20 mm were consolidated and used for ultrasonic testing, water capillary absorption test, and colour measurements. Cubes of 10 mm × 10 mm × 10 mm were consolidated and vacuum-impregnated in epoxy resin (Araldite^®^ 2020, Huntsman Advanced Materials, Basel, Switzerland) to produce polished sections for analysis with the scanning electron microscope. Polished surfaces were obtained by dry-cutting the embedded samples perpendicularly to the consolidated surface, and using a Buehler^®^ EcoMet^TM^ manual single grinder polisher (ITW Test & Measurement GmbH, Esslingen, Germany) equipped with silicon carbide paper discs and ethanol as a lubricant. 

### 2.2. Consolidants

Two nanomaterials were used in this study: nanolime and nanocalcite. The nanolime (NL) is a commercially-available product (Calosil^®^ E25) widely used for stone consolidation [49] and the consolidation of wall paintings surfaces [50]. The product is a suspension of nano-sized Ca(OH)_2_ crystals in ethanol. The concentration of lime is 25 g/L and the average particle size is 150 nm [51]. The product was supplied by IBZ-Salzchemie GmbH & Co.KG, Halsbruecke, Germany, and used as received according to instructions in the specification sheet.

The nanocalcite (NC) was a novel product developed by the ISTM centre of Florence (Italy), based on an aqueous dispersion of calcite nanoparticles (concentration 5% *w*/*w*, average size 30 nm) stabilised by an acrylic acid copolymer [19]. Its effectiveness as a potential consolidant was investigated in a preliminary work by Coltelli et al. [19], who reported uneven penetration within the porous network of the substrate, but also good adhesion to the substrate, effective bridges formation, and moderate shrinkage upon drying.

The di-ammonium hydrogen phosphate (reagent grade) was purchased in the form of fine grains from Carl Roth GmbH & Co.KG, Karlsruhe, Germany. A 1M solution was prepared by dissolving DAP in distilled water. This concentration was selected as previous studies showed that higher concentrations of DAP brought no further benefit [10,31].

### 2.3. Consolidation Treatments

Capillary suction was chosen over other application methods (i.e., brushing, poultice, or immersion) for two reasons: firstly, it allows mimicking conditions occurring during usual field application; and, secondly, it is more reproducible and this feature was privileged since a comparison of the consolidants effects was the aim of this study [52].

The consolidants were applied as individual products as well as in combinations, as reported in Table 1. The stone specimens were placed in Petri dishes filled with consolidant so that each sample was partially immersed in the consolidant up to about 5 mm, and let soak until the wetting front reached the top. Even if it was not fully soaked, after 1 h of absorption, the sample was removed from the Petri dish to reduce contact with the concentrated, viscous consolidant produced by the evaporation of the carrier or solvent. This, in fact, could have resulted in pores clogging and over-filling. Each application was repeated twice 24 h apart to improve the consolidant uptake. If an excess of consolidant was found on the treated surface, this was gently rubbed off with filter paper. As regards the combined treatments, one consolidant was applied first and, after complete drying, the second. Each consolidant was applied twice as described above, so that overall four applications were carried out on the sample. After treatment, the specimens were dried at 20 °C, 50% RH until constant weight and kept at the same conditions for 30 days before analysis to allow carbonation of the NL. To remove any soluble residue from inside the pores, samples treated with DAP were also rinsed with distilled water for three days, as outlined elsewhere [10]. Before and after treatment, all specimens were weighed to evaluate the uptake of the solid phase.

### 2.4. Evaluation of the Effectiveness and Compatibility of the Consolidation Treatments

Treated and untreated samples were tested according to the following methods.

*Micro-morphological characterisation and elemental analysis*. Uncoated polished sections of the treated samples were analysed using a Quanta^TM^ 250 FEG field-emission scanning electron microscope (SEM; Thermo Fisher Scientific, Waltham, MA, USA). Images were collected under a low vacuum at 20 kV acceleration voltage, using the backscattered electron (BSE) signal to visualise the consolidant inside the pore space by distinct grey values. Some of the collected images were post-processed using the Photoshop^®^ CC 2018 software (Adobe Inc., San Jose, CA, USA), and the consolidants precipitated inside the pore network were mapped by false-colouring. Other features of the micrographs (penetration depth, thickness, pore width, etc.) were measured using the software ImageJ v1.52i [53]. Particular attention was given to the following visual characteristics:

- microstructure of the consolidants (compactness, internal porosity, appearance, texture, and presence of shrinkage cracks);
- interaction of the consolidants with the stones (adhesion and induction of cracks to the substrate);
- binding capacity (formation of “bridges” of consolidant connecting grains of the stone, also defined as concave toroidal droplets; the formation thereof is known to have important implications for the performance of a consolidant [9]); and
- distribution of the consolidants inside the stone (penetration depth, smoothness of consolidation, and filling of pores).
- Elemental analysis was aimed at supporting the consolidants identification and was carried out using a TEAM^TM^ Pegasus energy-dispersive X-ray probe (EDX; Ametek EDAX, Berwyn, PA, USA) coupled to the SEM and interfaced by the Spectrum Viewer 4.0 software provided by the same company.

*Fourier-transform infrared spectroscopy (FT-IR)*. Chemical characterisation of precipitated products was carried out on some selected polished surfaces using a Lumos FT-IR microscope (Bruker, Billerica, MA, USA) in attenuated total reflectance (ATR) mode. Spectra were collected in the range 400–4000 cm^−1^ with 4 cm^−1^ resolution, from areas of interest (i.e., where an adequate amount of precipitated consolidant was visualised) previously identified by SEM. The collected spectra were then compared with reference materials for phase identification.

*Ultrasound pulse velocity test (UPV)*. Effectiveness of the treatments was assessed by measuring the UPV on sound, aged and treated specimens, according to the international standard ASTM C597-16 [54]. The equipment used was a CONSONIC C2-GS apparatus (Geotron-Elektronik, Pirna, Germany) interfaced by Light House Touch DW software, developed by the same company. Prior to the test, the specimens were measured using a Vernier calliper with 0.01 mm precision (Agar Scientific, Stansted, UK). Since the anisotropic structure of sedimentary stones affects UPV, for each sample one measurement was taken along the radial (R) direction and one along the longitudinal (L) direction, with respect to the bedding planes of the stone. UPV data of untreated specimens previously collected within the Nanocathedral project were used for this study. The average values of 130 sound, 130 aged Schlaitdorfer sandstone specimens, and 30 Auer limestone specimens were calculated, one measurement along both R and L directions for each specimen, and used as a reference value for the untreated lithotype.

*Capillary absorption test*. The test was performed following the procedure reported in the European standard EN15801 [55]. Water absorption coefficients (WACs) after 1 h were calculated for the sound, aged and treated specimens. Similar to UPV, water absorption data of untreated specimens previously collected were used. The average of WACs was measured on 45 sound Schlaitdorfer sandstone specimens and 15 aged Schlaitdorfer sandstone specimens, and 4 Auer limestone specimens were used as reference values for the untreated lithotypes.

*Colourimetry*. Colour measurements were carried out before and after treatment using a Sph850 spectrophotometer (ColorLite GmbH, Katlenburg-Lindau, Germany), according to the European standard EN15886 [56]. The average of three measured spots of each specimen was considered to account for possible natural variations in the stone colour. Spectra were collected in the range of 400–700 nm with a measuring spot of 4 mm^2^, under a D65 illuminant at 10° standard observer. The CIE (International Commission on Illumination) L*a*b* system was used, where L* (black-white), a* (red-green) and b* (yellow-blue) are spatial coordinates correlated to colour parameters. The colour difference before and after treatment was expressed by the Euclidean distance: ΔE* = √(Δa*)^2^ + (Δb*)^2^ + (ΔL*)^2^.

## 3. Results and Discussion

### 3.1. Uptake of Consolidant

The amount of solid phase precipitated in the pores was estimated gravimetrically after drying of the specimens. Relative weight increase values are reported in Table 2. Results show that, on average, a slightly higher amount of consolidant had deposited in the limestone than in the sandstone, in agreement with the expectation, considering the higher open porosity of the limestone. A significantly higher amount of consolidant had deposited after double-step treatments than after single-step treatments, in relation to the higher number of applications. During the treatments, it was observed that the absorption of NC and DAP solutions was easier than that of NL, which partially accumulated on the stone surface. NL absorption into the sandstone was slower compared to the limestone, and the surficial excess was thicker. The wet excess was readily rubbed off before drying. Some translucent efflorescences appeared on the surface after DAP treatments, but these were removed by the subsequent rinsing.

The capillary rise of the consolidants into the stone pore network can be reduced to the simplest case, described by the Jurin’s law (Equation (2)), that relates the liquid height (*h*) positively to the surface tension (*γ*) and inversely to the pore radius (*r*_0_) [57]:

(2) $$h=\frac{2\gamma \cos\theta }{\rho gr_{0}}.$$

According to Equation (2), the capillary rise of aqueous products is favoured over ethanol-based products as water has a higher surface tension than ethanol [58]. This may explain the higher weight increase observed in the sandstone samples treated with DAP and NC with respect to treatments with NL (see Table 2). Conversely, this behaviour was not observed in the limestone samples, where NL showed a higher weight increase than NC and DAP. As recently observed by Ban et al. [40], other phenomena related to the compositional and microstructural features of the stone fabric may influence the deposition process of consolidants, resulting in a weaker correlation between the absorbed and the precipitated consolidant. However, the specific characteristics (such as alkalinity, polarity of the Ca(OH)_2_ nanoparticles, particle size distribution, etc.) that allowed the NL to precipitate in such higher amount in the limestone have not been elucidated yet.

The effect of the order of application was also noteworthy. In the limestone, a greater weight increase was observed in DAP-NL than in NL-DAP treatment, possibly because, when NL was applied first, the DAP solution could not easily penetrate the stone. Such a difference was not observed for treatments with nanocalcite or in the sandstone samples, where all values are similar.

The recorded amount of precipitated consolidant was quite low for both lithotypes (wt% 0.5–1.7), even though, during treatment, the wetting front had reached the top of the specimens in most cases. This could have been due to a re-distribution of the consolidant particles, which were transported back towards the evaporation surface during drying of the carrier or solvent, as previously observed by Borsoi et al. [58]. A further reason may have been the early occlusion of pores through nanoparticles clustering and aggregation near the surface, as described by Baglioni et al. [59]. Moreover, HAP formation was limited by Ca^2+^ ions availability. It is possible that, once the calcareous outer layer of the pore walls had reacted, the remaining DAP could not reach further calcium ions, and thus the excess of DAP was transported out of the sample. Such a mechanism is in agreement with that described by Franzoni et al. [37].

### 3.2. Micro-Morphological Characterisation

**Figure 2:**
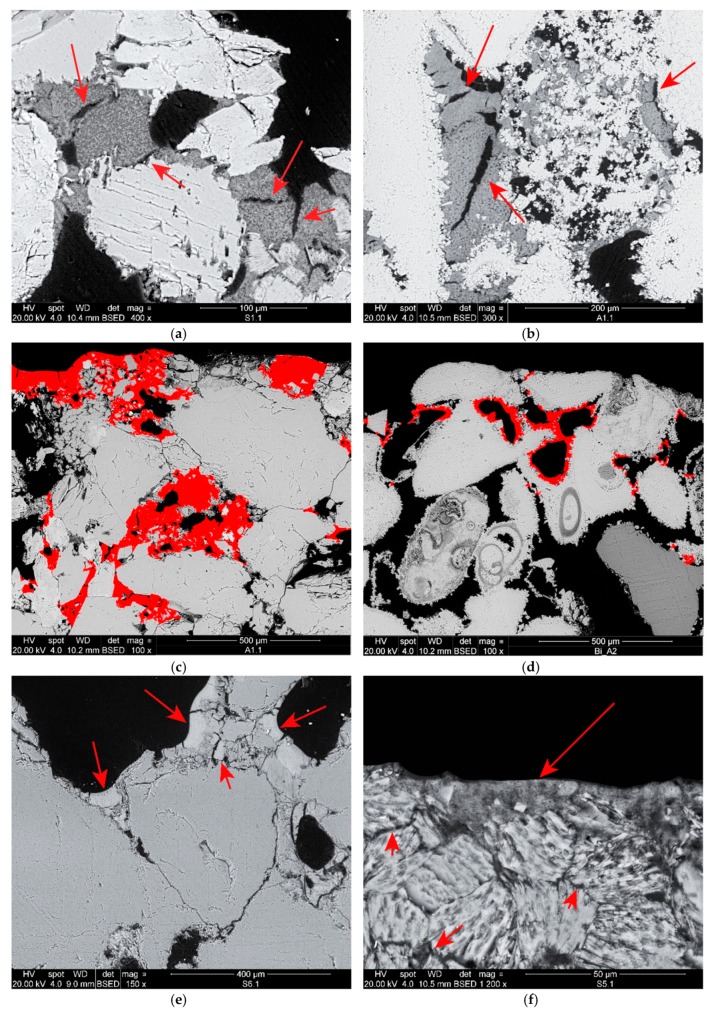
SEM micrographs of treated samples displaying some characteristics of the consolidants: adhesion, bridging capacity and porous microstructure with some shrinkage cracking (indicated by the arrows) of nanolime (NL) on (**a**) Schlaitdorfer sandstone and (**b**) Auer limestone; penetration depth (about 500 µm) of (**c**) NL on Schlaitdorfer sandstone and (**d**) Nanocalcite (NC) on Auer limestone with both consolidants shown in false colour; (**e**) adhesion and compact microstructure with some shrinkage cracks of di-ammonium phosphate-nanocalcite (DAP-NC) (see arrows) on Schlaitdorfer sandstone; and (**f**) adhesion of DAP-NC (see arrows) to the clayey matrix of Schlaitdorfer sandstone.

SEM micrographs showing some of the morphological characteristics of the consolidants are shown in Figure 2. The NL (Figure 2a–c) appeared unevenly distributed in the fabric of both lithotypes, as accumulations of product could be observed at the bottom and on the sides of the samples. The consolidant showed good grain adhesion in both stones, and bridges had efficiently formed between grains. Its micromorphology looked porous, with abundant shrinkage cracks (ranging 5–20 µm width), and an interface was visible within the newly formed layer suggesting diachronic precipitation due to the two applications. On the limestone, pores in the range 100–300 µm were partially filled with consolidant, resulting in a size reduction, whereas on the sandstone the consolidant was less evenly distributed, and the interstitial porosity of the clay matrix (<1 µm) was preferentially filled over bigger pores between quartz crystals.

The NC (Figure 2d) had a more compact and homogeneous micromorphology than the NL and fewer shrinkage cracks. As in the case of NL, most of the NC migrated to the evaporation surfaces, consolidating about 500 µm in depth of both lithotypes. On the limestone, bridges had effectively formed between grains, adhesion was very good, and pores were partially filled resulting in a reduction of their diameter. On the sandstone, the NC formed a thin layer scarcely adhered to the surface of the grains, and only few small bridges had formed. Similar to NL, the pores between interstitial clay minerals were preferentially filled over bigger pores.

**Figure 3:**
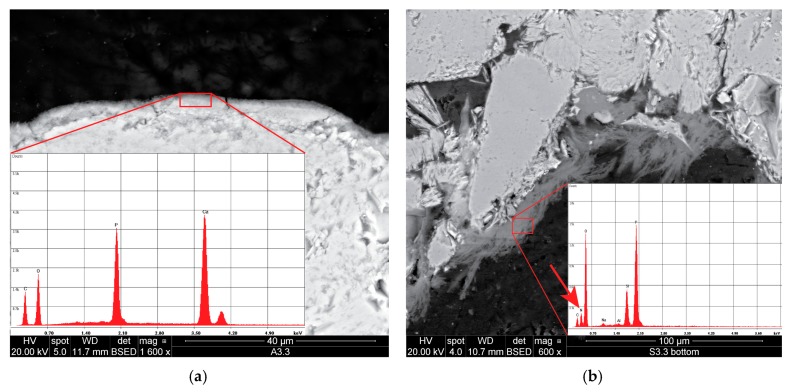
SEM micrographs of samples treated with DAP: (**a**) Phosphate layer formed on the surface of a limestone grain. In the inset, energy-dispersive X-ray (EDX) spectrum of such a layer showing strong P and Ca peaks. (**b**) Unreacted DAP found in the pores of the sandstone. In the inset, EDX spectrum showing, along with the P peak, presence of N (the relative peak is indicated by the arrow), which suggests that DAP had not converted into a Ca-P phase.

Regarding the phosphate treatments, application of DAP to the limestone resulted in the formation of a thin layer (ranging 2–5 μm thickness, Figure 3a) around the grains, produced by the reaction of DAP with the calcareous substrate. The layer showed a compact and homogeneous microstructure and appeared tightly adhered to the substrate so that the interface between the consolidant and the stone could be hardly identified. The Ca-P consolidant was not able to build bridges between the grains and filled only the micrometre-sized pores, forming a sort of film on their surface rather than producing a proper consolidating or binding action. This is probably due to the fact that the DAP conversion took place on the surface of the grains and, once this was covered with a dense layer of newly-precipitated Ca-P phase, the remaining DAP was not able to penetrate such a layer to gain access to further Ca^2+^ ions. 

**Figure 4:**
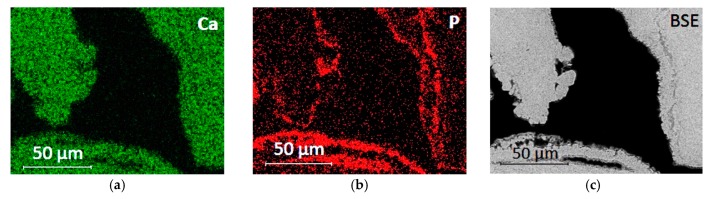
EDX elemental mapping of Auer limestone treated with DAP solution: (**a**) the map of calcium shows the calcareous stone; (**b**) the map of phosphorus shows the surface layer of the precipitated Ca-P phase; and (**c**) back-scattered electron image of the investigated area for comparison purpose.

The chemical composition of the phosphate-based consolidant was verified by EDX analysis, which revealed that Ca, P, and O were the dominant elements of this layer. The relative elemental maps are shown in Figure 4 together with the BSE image of the same area. The phosphate layer was found to be evenly distributed, up to about 2 cm in depth from the consolidated surface.

**Figure 5:**
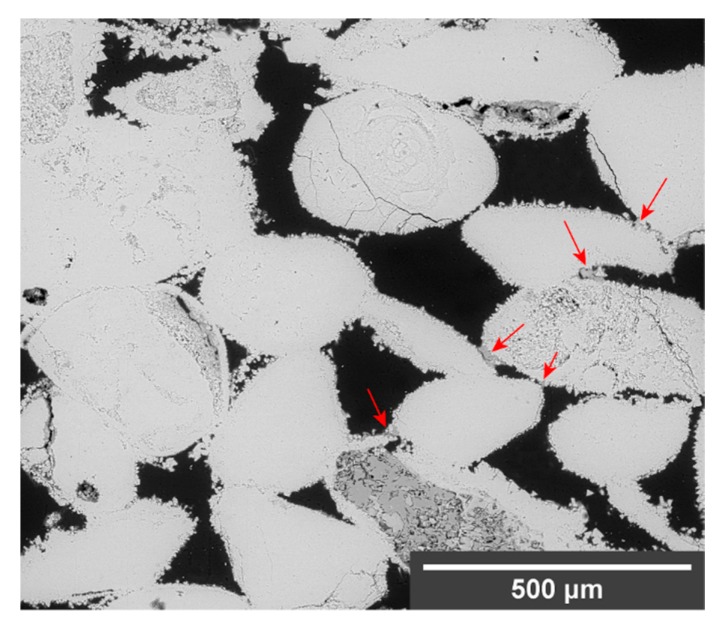
SEM micrograph of limestone treated with NL-DAP. Bridges of consolidant (indicated by the arrows) are found up to about 1 cm depth.

The combination of DAP with the nanosuspensions promoted bridges formation and penetration. The nanomaterials reached about 1 cm depth from the surface (Figure 5), in comparison with the few millimetres penetration obtained after the use of the neat products. In the treated limestone, two consolidation effects were observed: The first one entailed the formation of a layer similar to the one observed after treatment with neat DAP, characterised by a compact texture and a Ca:P ratio of about 1:1. The second one entailed the formation of small bridges with a more porous structure and a composition richer in calcium. These different consolidation effects were likely a consequence of the consolidants’ distinct behaviours: while DAP required conversion into HAP to accomplish a consolidation effect and was limited by the presence of a calcium-rich substrate, the nanosuspensions tended to form toroidal bridges between the stone grains upon the carrier evaporation. Furthermore, after treatments with NC-DAP, some of the bridges showed a granular texture. This was possibly a result of the reaction between DAP and the CaCO_3_ nanoparticles that, being dispersed in an aqueous medium, may have acted as nucleation centres for the newly formed Ca-P phase [60].

In the sandstone samples, unreacted DAP crystals were observed after treatment with neat DAP (Figure 3b), probably because of the limited amount of calcium ions, only available from the dolomitic fraction of the natural cement. It is possible that conversion to HAP did not reach completion and that residual DAP crystals were not completely removed by the rinsing. Differently, the combinations of DAP and nanosuspensions promoted the conversion reaction, producing a homogeneous Ca-P phase with abundant shrinkage cracks (Figure 2e,f). The consolidant formed bridges up to 20 μm wide, as well as a tightly-adhered Ca-P layer around the surface of the grains. As for consolidation with the nanosuspensions, the porosity of the interstitial clay matrix was preferentially filled over bigger pores. 

**Figure 6:**
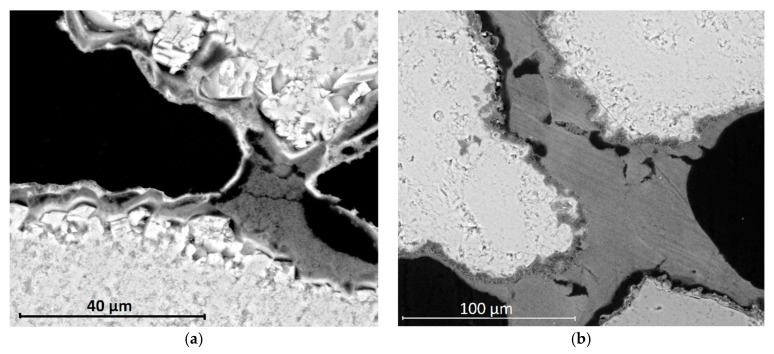
SEM micrographs displaying the effect of the order of application of the consolidants in the limestone: (**a**) after treatment NL-DAP, stone grains are linked by a bridge of nanolime, surrounded by a detached, compact phosphate layer; and (**b**) after treatment DAP-NL, stone grains are surrounded by a well-adhered phosphate layer and linked by a bridge of nanolime.

The effect of the application sequence (e.g., NL-P and P-NL) was also taken into consideration, and similar results were observed between the two lithotypes. When a nanomaterial was applied before DAP, the typical morphology observed is shown in Figure 6a. In these cases, bridges between the stone grains were probably formed during the first step and the subsequent application of the DAP resulted in a detached layer which unlikely added a consolidation effect to the treatment. Conversely, after the treatments initiated with the DAP, the phosphate layer covered the stone grains, and the toroidal bridges that formed in the second step appeared better adhered to the grains (Figure 6b).

Overall, the limestone treated with nanosuspensions showed better morphological features than the sandstone. This is probably due to the similar composition between the consolidants and the limestone, which allowed a stronger interaction than with silicate-based substrates [61]. In the sandstone, a tight adhesion was only observed to the interstitial dolomitic and clayey binder. Some of the observed advantages of the NC over NL (less shrinkage, tighter adhesion) were probably due to: (i) the smaller particle size of NC, which entail a higher reactivity and therefore the ability to establish more interactions with the substrate (i.e., the pore walls); (ii) the different carrier and evaporation kinetics, as water dries more slowly than ethanol and may limit shrinkage phenomena [13]; and (iii) the fact that NC is already in the calcite form, whereas NL undergoes carbonation through a series of dissolution/re-precipitation steps [62], which might compromise the adhesion to the substrate and foster the development of cracks. NC also showed good bridging capacities, as previously reported by Coltelli et al. [19]. Both nanosuspensions produced their consolidating effects by replacing the lost natural cement through bridges formation. Conversely, hydroxyapatite precipitated as a layer covering calcium-rich substrates. Considering the high stability and resistance to acid attacks of this mineral [63], it is possible that DAP treatments may result in a sort of passivation layer on the pore surfaces with a protective function rather than accomplishing an actual consolidation action [64]. Such a passivating effect should be further verified, but it has been already observed and exploited for the protection of deteriorated marble in previous studies [65].

### 3.3. Reaction Products Composition

The conversion of DAP into HAP was investigated using the FT-IR spectroscopy on polished sections of treated limestone. All collected spectra showed calcium carbonate and hydroxyapatite as main compounds and a minor fraction of organic material, most likely due to the embedding epoxy resin. Characteristic primary peaks in the collected spectra are listed in Table 3. Ammonium bands (2400–3200 cm^−1^) were not detected in any of the investigated samples, suggesting that the amount of unreacted ammonium salt inside the stone pores was below the detection limit of the equipment used. Calcium hydroxide produces a strong, sharp peak in the O-H region (3640 cm^−1^ [66]). Such a peak could not be observed in any of the samples, and no difference in the O-H region was observed between spectra collected on samples treated with NL and those treated with NC or only DAP, suggesting that the NL mostly converted into calcium carbonate upon contact with air. 

**Figure 7:**
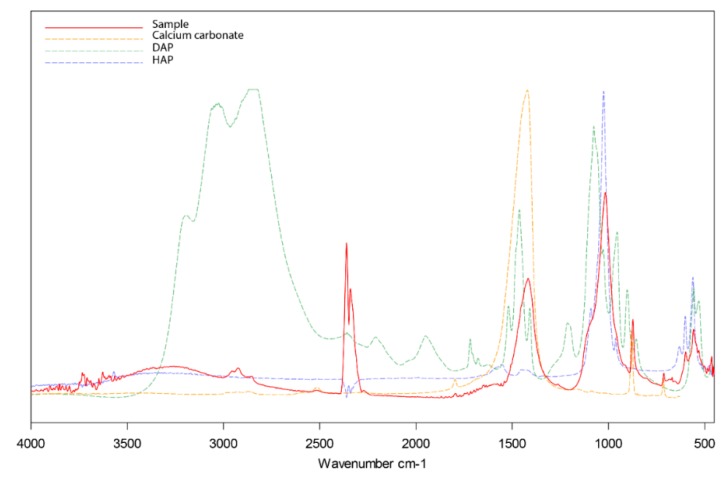
Fourier-transform infrared (FT-IR) spectra of sample of limestone treated with DAP (solid red line), pure calcium carbonate (dashed yellow line), pure DAP (dashed green line), and hydroxyapatite (dashed blue line).

To corroborate the conversion of the applied DAP into HAP, the spectra were compared with the reference spectra of calcium carbonate, DAP and hydroxyapatite, as shown in Figure 7. Although the results suggest that the DAP conversion took place in all analysed samples, previous studies suggested that the reaction products were most probably a mixture of HAP, octacalcium phosphate and other insoluble precursors [10,32]. According to the same studies, these compounds are expected to eventually transform into the most thermodynamically stable HAP through a series of dissolution and reprecipitation processes. Clear identification of these phases was hindered by the wide overlapping of their characteristic bands with those of the substrate. Furthermore, the precipitation mechanisms of hydroxyapatite and related precursors are complex and not yet fully understood, and their identification can be challenging not only by FT-IR spectroscopy but also using other techniques [32].

### 3.4. Effectiveness of the Treatments

UPV measurements were carried out on sound, aged and treated specimens to assess the effectiveness of the treatments. The measured velocities and their relative increase values are listed in Table 4. UPV values of the untreated samples were similar to those reported in the literature for sound Auer limestone (2.6 km/s), sound Schlaitdorfer sandstone (3.8 km/s) and aged Schlaitdorfer sandstone (1.3 km/s) [41,44,46]. Although little effects were observed between the various treatments, the two lithotypes remarkably showed different responses to the consolidation: while the limestone showed a slight UPV increase after the treatments, the sandstone produced more evident UPV increases (mostly >100%), regaining a value close to the natural sound stone. Furthermore, treatments based on DAP-nanosuspension combinations produced higher UPV increase than those based on neat products, possibly as a result of a larger amount of absorbed consolidant. The highest increase was obtained after treatment with DAP-NC. However, no straightforward relationship was evident between the amount of the absorbed consolidant (Table 2) and the UPV increase. UPV values of the consolidated, aged sandstone were lower than the value of the sound stone, suggesting that the treatments are unlikely able to induce abrupt variations in mechanical properties of the treated stone. According to Delgado Rodrigues et al. [67], who assessed the incompatibility risk of a treatment based on the per cent increase in mechanical properties between treated and untreated material, a low risk can be assigned to all treatments on the sandstone (0%, or less, increase with respect to the sound stone), and a low-to-medium risk to the limestone (about 10% increase). According to the same study, higher increase (>50%) would be classified as high risk.

The different response to the treatments of the two lithotypes has been likely affected by some initial characteristics of the stone fabrics. These may have influenced the consolidants performance and their effect on the pore size distribution, as it had been previously observed by other authors [45,68]. HAP and NL are reported to cause a substantial reduction in coarser pores (>1 µm), and an increase in finer pores (<0.1 µm), as a consequence of pore size reduction and newly-formed inner porosity of the precipitated consolidants [10,28,29]. This was observed by SEM only in the limestone, where the precipitation of both the nanosuspensions and the phosphate layer had reduced the diameter of coarser pores. Conversely, the porosity of the interstitial clay matrix (<1 µm) in the sandstone was preferentially consolidated over bigger pores among quartz crystals (Figure 2a,c,f). This behaviour was also observed by Ban et al. [45], who claimed that preferential strengthening of the intergranular clay minerals occurred and was accountable for strength increase. The role of clay minerals in the mechanical properties of stones has been thoroughly investigated and the consolidation of clay-bearing stones is a well-known issue [69,70,71,72,73]. Hence, the abundance of clay minerals in the sandstone, present as a filler within the stone fabric, could have been the reason, or one of the reasons, the UPV increase after the treatments was higher in the sandstone than in the limestone. Although further investigations are needed to support this interpretation, it was clear that the initial characteristics of the stones played a key role in the consolidants behaviour and performance, and therefore such characteristics should always be investigated and considered before designing any consolidation treatment.

### 3.5. Effects of Treatments on Water Transport Properties

The capillary absorption test was carried out to obtain information about the effects of treatments on water transport properties, a key parameter when considering the compatibility of a consolidation method. The water absorption coefficients at 1 h (WACs) of treated and untreated samples and their relative increase are reported in Table 5. Similar to the UPV results, different responses were recorded between the two lithotypes. The untreated stones had already different water transport properties: the sound limestone could absorb about 1.6 times more than the aged sandstone. After the treatments, the limestone sorptivity was only slightly altered, with values fluctuating around 0% WAC increase, whereas the sandstone sorptivity had considerably reduced, with an average WAC decrease of about 45%.

These results show a similar trend to that observed after UPV tests, confirming that the initial characteristics of the stones, possibly pore size distribution and the presence of clay minerals, played a major role in determining the effect of the treatments, rather than the type of consolidant used.

Regarding the treatments’ compatibility, the movement of liquid water within the sample was not completely blocked after any of the treatments. It is widely accepted that a consolidant should not drastically alter the water transport properties nor arrest the water exchange between the stone and the environment. Generally, treatments based on inorganic products are considered more compatible than those based on polymers or alkoxysilanes, which have shown to excessively reducing liquid-water movements, especially in highly porous stones [7]. According to the indicators of incompatibility established by Delgado Rodrigues et al. [67], both HAP and the nanosuspensions could be considered as low-risk treatments with the tested limestone (sorptivity had reduced by about 10%, or less). Conversely, sorptivity had reduced by 30–60% in the sandstone, thus ranking as a medium risk of incompatibility. Although complete pore occlusion had not occurred, the substantial reduction in sorptivity may hinder water transport and slower the drying rate, increasing the risk of detrimental consequences to the stone.

### 3.6. Aesthetical Compatibility of Treatments

Colour measurements were performed to evaluate the aesthetical compatibility of the consolidants with the tested substrates. The results of this test are illustrated in Table 6, where the overall difference (ΔE*) defined at the end of Section 2.4 and the differences of the single coordinates (ΔL*, Δa*, Δb*) between the treated and untreated stones are reported.

The selected threshold used to discriminate between acceptable and unacceptable treatments is a crucial step when assessing conservation treatments. Generally, only ΔE* < 5 is used in the conservation field [74] but other values might as well be taken into account, such as the threshold of discernibility by naked eye (ΔE* > 2.3) [75]. A helpful grading system was provided by Delgado Rodrigues et al. [67], who defined colour variations on stone surfaces according to their risk of incompatibility. Table 7 shows the tested treatments, sorted according to the criteria suggested by these authors. 

It is remarkable that most DAP-based treatments fall into the high-risk category. High colour variations after DAP treatments were also reported by Sassoni et al. [10], who measured a ΔL* increase responsible for a variation over the general acceptance threshold. However, the authors in the same study pointed out that colour differences due to the treatments were less significant than differences due to the natural variability of the stone appearance. This was also the case for the tested lithotypes. Indeed, higher colour differences were measured between the untreated samples (ΔE* = 6.39 and ΔE* = 11.12 for limestone and sandstone, respectively, determined as the average of 16 measurements) than between treated and untreated samples. The highest colour differences were recorded after P-NC treatments on both lithotypes, respectively ΔE* = 6.2 and ΔE* = 8.7.

It is worth noting that highly heterogeneous materials, such as coarse and porous stones, may tolerate higher colour variations without impairing the overall appearance and that other characteristics of the treated stone, such as variations in surface roughness and glossiness, should also be taken into account when evaluating the visual impact of conservation treatments [52,76]. The treatment procedure, e.g., the application method, is a further factor likely to induce variations in stone colour. This aspect was investigated by Ferreira Pinto et al. [52], who concluded that the brushing technique should be selected if the aim is to evaluate the visual impact of consolidants. Further studies should consider whether aesthetical compatibility of DAP applications may be improved by modifying the testing protocol. Monitoring of the treated surfaces should also be taken into consideration as long-term exposure might attenuate the colour changes. 

## 4. Conclusions

The aim of this study was the evaluation of the effectiveness and compatibility of consolidation treatments based on various combinations of di-ammonium phosphate (DAP) solution and two calcium-based nanosuspensions, a nanolime (NL) and a nanocalcite (NC), applied to Schlaitdorfer sandstone and Auer limestone. 

The treatments were able to improve the compactness of the stones they were applied to. However, the stone textural and chemical characteristics played a predominant role in the performance of the consolidants, as the substrate’s effect was greater than the effect of the treatments themselves. Overall, they produced a greater impact on the Schlaitdorfer sandstone than on the Auer limestone in terms of UPV and WAC. As regards physical compatibility, the treatments could be assessed with a low risk of incompatibility on the limestone, as water sorption had not dramatically reduced after application of the consolidants. On the other hand, a substantial reduction in water sorption was observed on the sandstone after the consolidants’ application, meaning a medium risk of incompatibility. Nanoconsolidants and DAP should thus be applied with caution on similar lithotypes, especially if high moisture is an issue of the treated object.

As regards the application of pure nanoconsolidants, their main issue concerned the limited penetration into the stone which reached a depth of about 0.5 mm. Such penetration can be evaluated in relation to the depth of the layer affected by the deterioration process, which typically varies from a few centimetres in porous stones to less than a millimetre in marbles. This suggests that nanomaterials could be successfully employed for the consolidation of fine-grained, compact stones affected by decohesion processes limited to the very surface. A further potential use could be the pre-consolidation of stones in an advanced deterioration state, requiring an immediate intervention to secure some initial surface cohesion. In such a case, the tested nanomaterials would be advantageous as they do not alter surface properties, allowing their retreatment. Moreover, in the case of NC, the consolidation effect is accomplished in short time after application (drying time, i.e., usually less than 48 h), whereas other common consolidants require several weeks to complete curing. The NC could also be assessed with low risk of aesthetical incompatibility on both stones, whereas the NL should be used with caution on the Schlaitdorf sandstone as slight yellowing might occur after treatment.

The application of pure DAP showed to be effective on the limestone, where most of the salt was able to react and convert into stable hydroxyapatite, well adhered to the grains’ surface. On the sandstone, residues of unconverted DAP were observed, hence a further source of Ca^2+^ ions is necessary, e.g., a subsequent application of a calcium-based consolidant. However, the presence of possible unreacted DAP residues and the detailed characterisation of the reaction products should be further clarified to avoid the introduction of detrimental soluble salts into the stone upon treatment.

The combined application of the nanoconsolidants and DAP showed to produce two different morphologies, one produced by hydroxyapatite precipitation in the form of a thin, compact layer on the grains’ surface, and the other produced by the deposition of the nanoparticles in the form of toroidal bridges linking the grains. The combined treatments showed a greater effectiveness than those based on neat products, as a result of a more effective pore filling and a higher penetration depth of about 1 cm. Moreover, DAP is recommended to be applied first and then the nanoconsolidant, as a stronger adhesion to the substrate was obtained than through the reverse order of application. 

A limitation of the combined treatments highlighted by this study was the high risk of aesthetical compatibility as a result of yellowing of the surface after applications. The treatments should thus only be applied to stones that naturally show high colour variability, where colour variations brought by the treatments become less significant. It should also be taken into account that effects of long-term exposure likely attenuate such risk and this is one of the reasons a monitoring schedule after any consolidation intervention is of uttermost importance.

Finally, although it should be stressed that field conditions are usually hardly controllable and that unexpected responses to treatments are likely to be found, preliminary evaluation is a crucial step in the selection of a consolidation treatment. In such context, laboratory-based trials carried out in controlled and reproducible conditions allow for an initial evaluation of the consolidants performance.

## Figures and Tables

**Table 1:** Set of tested consolidation treatments.

Treatment	Step 1	Step 2
NL	Nanolime	-
NC	Nanocalcite	-
DAP	Di-ammonium phosphate	-
NL-DAP	Nanolime	Di-ammonium phosphate
NC-DAP	Nanocalcite	Di-ammonium phosphate
DAP-NL	Di-ammonium phosphate	Nanolime
DAP-NC	Di-ammonium phosphate	Nanocalcite

**Table 2:** Relative weight increase (%) of the stone specimen after consolidation treatment and complete drying. The average weight increase ($\bar{\mathrm{wt}}$%) and standard deviations for the one-step treatments and the two-steps treatments are reported for each lithotype.

Stone Type	NL	NC	DAP	$\bar{\mathrm{wt}}\%$	NL-DAP	NC-DAP	DAP-NL	DAP-NC	$\bar{\mathrm{wt}}\%$
Schlaitdorfer Sandstone	0.2	0.4	1.0	0.5 ± 0.4	1.2	1.2	1.1	1.4	1.2 ± 0.1
Auer Limestone	1.5	0.6	0.6	0.9 ± 0.5	1.4	1.3	2.1	1.6	1.7 ± 0.4

**Table 3:** Assignment of the main peaks in the Fourier-transform infrared (FT-IR) spectrum of Auer limestone treated with di-ammonium phosphate (DAP).

Wavenumber (cm^−1^)	Assignment	Component
2924	C-H stretching vibration	Epoxy resin
2518	I overtone CO_3_^2−^ stretching	Calcium carbonate
2349	O=C=O stretching vibration	Atmospheric CO_2_
1799	I overtone CO_3_^2−^ in-plane bending	Calcium carbonate
1397	CO_3_^2−^ stretching vibration	Calcium carbonate
1022	PO_4_^3−^ stretching vibration	Hydroxyapatite
868	CO_3_^2−^ out-of-plane bending	Calcium carbonate
713	CO_3_^2−^ in-plane bending	Calcium carbonate
600	PO_4_^3−^ in-plane bending	Hydroxyapatite
560	PO_4_^3−^ out-of-plane bending	Hydroxyapatite

**Table 4:** UPV (km/s) and UPV relative increase (%) values for untreated and treated samples of Schlaitdorfer sandstone and Auer limestone, measured in the radial (R) and longitudinal (L) directions.

Treatment	UPV (R, km/s)	UPV (L, km/s)	UPV (R, km/s)	UPV (L, km/s)	UPV Increase (R, %)	UPV Increase (L, %)	UPV Increase (R, %)	UPV Increase (L, %)
	Schlaitdorfer Sandstone		Auer Limestone		Schlaitdorfer Sandstone ^1^		Auer Limestone ^1^	
Sound	3.86 ± 0.17	3.47 ± 0.17	2.68 ± 0.13	2.44 ± 0.13	-	-	-	-
Aged	1.43 ± 0.18	1.20 ± 0.15	-	-	-	-	-	-
NL	1.85	1.74	2.92	2.77	29	45	9	14
NC	2.27	2.05	2.86	2.73	59	71	7	12
DAP	3.34	2.00	3	2.74	134	67	12	12
NL-DAP	2.87	2.66	3.1	3	101	122	16	23
NC-DAP	2.87	2.69	3.06	2.92	101	124	14	20
DAP-NL	3.03	2.88	2.97	2.76	112	140	11	13
DAP-NC	3.39	3.35	2.93	2.73	137	179	9	12

^1^ For Schlaitdorfer sandstone, the UPV increase was calculated comparing the values of the treated samples with the aged untreated samples; for Auer limestone, with the sound untreated samples.

**Table 5:** Water absorpion coefficients (WACs) of Schlaitdorfer sandstone and Auer limestone for untreated and treated specimens and relative WAC increase post-consolidation.

Treatment	WAC (kg/m^2^⋅h^0.5^)		WAC Increase ^1^ (%)	
	Schlaitdorfer Sandstone	Auer Limestone	Schlaitdorfer Sandstone	Auer Limestone
Sound	3.25 ± 0.86	9.31 ± 0.3	-	-
Aged	5.91 ± 1.24	-	-	-
NL	3.62	8.87	−39	−5
NC	3.36	9.62	−43	3
DAP	2.90	8.78	−51	−6
NL-DAP	3.06	8.99	−48	−3
NC-DAP	2.61	9.3	−56	0
DAP-NL	3.10	8.1	−48	−13
DAP-NC	3.98	8.56	−33	−8

^1^ For Schlaitdorfer sandstone, the WAC increase was calculated comparing the values of the treated samples with the aged untreated samples; for Auer limestone, with the sound untreated samples.

**Table 6:** Colour differences (Δ) between treated and untreated stone.

Treatment	Schlaitdorfer Sandstone				Auer Limestone			
	ΔL*	Δa*	Δb*	ΔE*	ΔL*	Δa*	Δb*	ΔE*
NL	−1.4	2.9	−0.7	3.3	−1.4	−0.2	1.1	1.8
NC	1.9	0.2	1.4	2.3	1.1	−0.5	0.7	1.4
DAP	−2.9	0.7	4.9	5.7	1.7	−0.5	2.3	2.9
NL-DAP	−4.8	2.1	6.8	8.6	−0.6	0.1	5.8	5.8
NC-DAP	−3.6	0.4	6.4	7.3	−0.9	0.2	4.8	4.9
DAP-NL	−4.1	0.7	4.7	6.3	−0.2	−0.3	5.5	5.5
DAP-NC	−7.0	0.8	5.2	8.7	−0.1	−0.1	6.2	6.2

**Table 7:** Aesthetical compatibility of the treatments, according to the criteria outlined by [67].

Risk of Incompatibility	Colour Difference	Treatments	
		Sandstone	Limestone
Low	ΔE* < 3	NC	NL, NC, DAP
Medium	3 < ΔE* < 5	NL	NC-DAP
High	ΔE* > 5	DAP, NL-DAP, NC-DAP, DAP-NL, DAP-NC	NC-DAP, DAP-NL, DAP-NC

## References

[1] Verges-Belmin V. (2008). Illustrated Glossary on Stone Deterioration Patterns.

[2] Ente Nazionale Italiano di Unificazione (2006). UNI 11182: Beni Culturali: Materiali Lapidei Naturali ed Artificiali: Descrizione Della Forma di Alterazione—Termini e Definizioni.

[3] Siegesmund S., Weiss T., Vollbrecht A. (2002). Natural Stone, Weathering Phenomena, Conservation Strategies and Case Studies.

[4] Tiano P. (2002). Biodegradation of Cultural Heritage: Decay Mechanisms and Control Methods. Proceedings of the 9th International Workshop ARIADNE—Historic Materials and Their Diagnostics.

[5] Winkler E.M. (1994). Stone in Architecture.

[6] Price C.A. (1996). Stone Conservation: An Overview of Current Research.

[7] Odgers D., Henry A. (2012). English Heritage Practical Building Conservation Stone.

[8] Amoroso G.G. (2002). Trattato di Scienza Della Conservazione dei Monumenti.

[9] Scherer G.W., Wheeler G.S. (2009). Silicate Consolidants for Stone. Key Eng. Mater..

[10] Sassoni E., Naidu S., Scherer G.W. (2011). The use of hydroxyapatite as a new inorganic consolidant for damaged carbonate stones. J. Cult. Herit..

[11] Rodriguez-Navarro C., Suzuki A., Ruiz-Agudo E. (2013). Alcohol dispersions of calcium hydroxide nanoparticles for stone conservation. Langmuir.

[12] Lazzarini L., Laurenzi Tabasso M. (2010). Il Restauro Della Pietra.

[13] Hansen E., Doehne E., Fidler J., Larson J., Martin B., Matteini M., Rodriguez-Navarro C., Pardo E.S., Price C., de Tagle A. (2003). A review of selected inorganic consolidants and protective treatments for porous calcareous materials. Stud. Conserv..

[14] Sasse H.R., Honsinger D., Schwamborn B. (1993). New technology in porous stone conservation. Conservation of Stone and Other Materials: Proceedings of the International RILEM/UNESCO Congress.

[15] Giorgi R., Baglioni M., Berti D., Baglioni P. (2010). New Methodologies for the Conservation of Cultural Heritage: Micellar Solutions, Microemulsions, and Hydroxide Nanoparticles. Acc. Chem. Res..

[16] Balliana E., Ricci G., Pesce C., Zendri E. (2016). Assessing the value of green conservation for cultural heritage: Positive and critical aspects of already available methodologies. Int. J. Conserv. Sci..

[17] Borgioli L. (2002). Polimeri di Sintesi per la Conservazione Della Pietra.

[18] Perez-Ema N., de Buergo M.A., Bustamante R., Gomez-Heras M. (2015). Changes in Petrophysical Properties of the Stone Surface due to Past Conservation Treatments in Archaeological Sites of Merida (Spain). Eng. Geol. Soc. Territ..

[19] Coltelli M.-B., Paolucci D., Castelvetro V., Bianchi S., Mascha E., Panariello L., Pesce C., Weber J., Lazzeri A. (2018). Preparation of water suspensions of nanocalcite for cultural heritage applications. Nanomaterials.

[20] Sassoni E., Graziani G., Franzoni E. (2016). An innovative phosphate-based consolidant for limestone. Part 2: Durability in comparison with ethyl silicate. Constr. Build. Mater..

[21] Barberio M., Veltri S., Imbrogno A., Stranges F., Bonanno A., Antici P. (2015). TiO_2_ and SiO_2_ nanoparticles film for cultural heritage: Conservation and consolidation of ceramic artifacts. Surf. Coat. Technol..

[22] De Rosario I., Elhaddad F., Pan A., Benavides R., Rivas T., Mosquera M.J. (2015). Effectiveness of a novel consolidant on granite: Laboratory and in situ results. Constr. Build. Mater..

[23] De Ferri L., Lottici P.P., Lorenzi A., Montenero A., Salvioli-Mariani E. (2011). Study of silica nanoparticles—Polysiloxane hydrophobic treatments for stone-based monument protection. J. Cult. Herit..

[24] Dei L., Salvadori B. (2006). Nanotechnology in cultural heritage conservation: Nanometric slaked lime saves architectonic and artistic surfaces from decay. J. Cult. Herit..

[25] Borsoi G., Veiga R., Silva A.S. (2013). Effect of nanostructured lime-based and silica-based products on the consolidation of historical renders. HMC13: Proceedings of the 3rd Historic Mortars Conference.

[26] Slížková Z., Frankeová D., Drdácký M. (2013). Strengthening of poor lime mortar with consolidation agents. HMC13: Proceedings of the 3rd Historic Mortars Conference.

[27] Zornoza-Indart A., López-Arce P., Gómez-Villalba L.S., Varas-Muriel M.J., Fort R. (2012). Consolidation of Deteriorated Carbonate Stones with Ca(OH)_2_ Nanoparticles. Proceedings of the 12th International Congress on the Deterioration and Conservation of Stone.

[28] Costa D., Delgado Rodrigues J. (2012). Consolidation of a Porous Limestone with Nanolime. Proceedings of the 12th International Congress on the Deterioration and Conservation of Stone.

[29] Pesce G.L., Morgan D., Odgers D., Henry A., Allen M., Ball R.J. (2013). Consolidation of weathered limestone using nanolime. Proc. Inst. Civ. Eng. Constr. Mater..

[30] Duchêne S., Detalle V., Favaro M., Ossola R., Tomasin P., De Zorzi C., El Habra N. (2012). Nanomaterials for the consolidation of marble and wall paintings. Proceedings of the 12th International Congress on the Deterioration and Conservation of Stone.

[31] Sassoni E., Franzoni E., Pigino B., Scherer G.W., Naidu S. (2013). Consolidation of calcareous and siliceous sandstones by hydroxyapatite: Comparison with a TEOS-based consolidant. J. Cult. Herit..

[32] Possenti E., Colombo C., Bersani D., Bertasa M., Botteon A., Conti C., Lottici P.P., Realini M. (2016). New insight on the interaction of diammonium hydrogenphosphate conservation treatment with carbonatic substrates: A multi-analytical approach. Microchem. J..

[33] Matteini M., Rescic S., Fratini F., Botticelli G. (2011). Ammonium phosphates as consolidating agents for carbonatic stone materials used in architecture and cultural heritage: Preliminary research. Int. J. Archit. Herit..

[34] Kumar H., Behal I. (2017). Volumetric and ultrasonic properties of α-amino acids (glycine, L-alanine and L-valine) in aqueous diammonium hydrogen phosphate at different temperatures and concentrations. J. Mol. Liq..

[35] Mathew M., Takagi S. (2012). Structures of biological minerals in dental research. J. Res. Natl. Inst. Stand. Technol..

[36] Naidu S., Scherer G.W. (2014). Nucleation, growth and evolution of calcium phosphate films on calcite. J. Colloid Interface Sci..

[37] Franzoni E., Sassoni E., Graziani G. (2015). Brushing, poultice or immersion? The role of the application technique on the performance of a novel hydroxyapatite-based consolidating treatment for limestone. J. Cult. Herit..

[38] Naidu S., Liu C., Scherer G.W. (2015). Hydroxyapatite-based consolidant and the acceleration of hydrolysis of silicate-based consolidants. J. Cult. Herit..

[39] Sassoni E. (2018). Hydroxyapatite And Other calcium phosphates for the conservation of cultural heritage: A review. Materials.

[40] Ban M., De Kock T., Ott F., Barone G., Rohatsch A. (2018). Neutron Radiography Study of Laboratory Ageing and Treatment Applications with Stone Consolidants. Nanomaterials.

[41] Graue B.J. (2013). Stone Deterioration and Replacement of Natural Building Stones at Cologne Cathedral A Contribution to the Preservation of Cultural Heritage. Ph.D. Thesis.

[42] Lazzeri A., Coltelli M.-B., Castelvetro V., Bianchi S., Chiantore O., Lezzerini M., Niccolai L., Weber J., Rohatsch A., Gherardi F. (2016). European Project NANO-CATHEDRAL: Nanomaterials for Conservation of European Architectural Heritage Developed by Research on Characteristic Lithotypes. Proceedings of the 13th International Congress on the Deterioration and Conservation of Stone.

[43] Grimm W.D., Petzet M. (1990). Bildatlas Wichtiger Denkmalgesteine der Bundesrepublik Deutschland.

[44] Ban M., Baragona A., Ghaffari E., Weber J., Rohatsch A. (2016). Artificial aging techniques on various lithotypes for testing of stone consolidants. Proceedings of the 13th International Congress on the Deterioration and Conservation of Stone.

[45] Ban M., Mascha E., Weber J., Rohatsch A. (2019). Efficiency and Compatibility of Selected Alkoxysilanes on Porous Carbonate and Silicate Stones. Materials.

[46] Rohatsch A. (2005). Neogene Bau-un Dekorgesteine Niederösterreichs un des Burgenlandes.

[47] Moshammer B., Uhlir C., Rohatsch A., Unterwurzacher M. (2015). Adnet ‘marble’, Untersberg ‘marble’and Leitha limestone—Best examples expressing Austria’s physical Cultural Heritage. Eng. Geol. Soc. Territ..

[48] Gradstein F.M., Ogg J.G., Hilgen F.J. (2012). On the Geologic Time Scale. Newsl. Stratigr..

[49] Macounová D., Bayer K., Slížková Z., Weber J., Navrátilová M., Ghaffari E., Hvězda D. (2016). NANOLITH—Konservierung von Leithakalken auf Basis von Calciumhydroxid-Nanopartikeln.

[50] Chelazzi D., Poggi G., Jaidar Y., Toccafondi N., Giorgi R., Baglioni P. (2013). Hydroxide nanoparticles for cultural heritage: Consolidation and protection of wall paintings and carbonate materials. J. Colloid Interface Sci..

[51] Safety Data Sheet According to Regulation (EG) No. 1907/2006. https://ibz-freiberg.de/downloads/pdf/produkte/sd/eng/msds_Calosil-E.pdf.

[52] Ferreira Pinto A.P., Delgado Rodrigues J. (2014). Impacts of consolidation procedures on colour and absorption kinetics of carbonate stones. Stud. Conserv..

[53] Schneider C.A., Rasband W.S., Eliceiri K.W. (2012). NIH Image to ImageJ: 25 years of image analysis. Nat. Methods.

[54] ASTM International (2016). ASTM C597-16, Standard Test Method for Pulse Velocity Through Concrete.

[55] The European Committee for Standardization (CEN) (2010). Standard EN 15801, Conservation of Cultural Property—Test Methods—Determination of Water Absorption by Capillarity.

[56] The European Committee for Standardization (CEN) (2010). Standard EN 15886, Conservation of Cultural Property—Test Methods—Colour Measurement of Surfaces.

[57] de Gennes P.-G., Brochard-Wyart F., Quéré D. (2004). Capillarity and Gravity. Capillarity and Wetting Phenomena.

[58] Borsoi G., Lubelli B., van Hees R., Veiga R., Silva A.S. (2016). Understanding the transport of nanolime consolidants within Maastricht limestone. J. Cult. Herit..

[59] Baglioni P., Chelazzi D., Giorgi R., Carretti E., Toccafondi N., Jaidar Y. (2014). Commercial Ca(OH)_2_ nanoparticles for the consolidation of immovable works of art. Appl. Phys. A Mater. Sci. Process..

[60] Xia X., Chen J., Shen J., Huang D., Duan P., Zou G. (2018). Synthesis of hollow structural hydroxyapatite with different morphologies using calcium carbonate as hard template. Adv. Powder Technol..

[61] Otero J., Starinieri V., Charola A.E. (2018). Nanolime for the consolidation of lime mortars: A comparison of three available products. Constr. Build. Mater..

[62] Rodriguez-Navarro C., Kudłacz K., Cizer Ö., Ruiz-Agudo E. (2015). Formation of amorphous calcium carbonate and its transformation into mesostructured calcite. CrystEngComm.

[63] Kalita S.J., Bhardwaj A., Bhatt H.A. (2007). Nanocrystalline calcium phosphate ceramics in biomedical engineering. Mater. Sci. Eng. C.

[64] Ferreira Pinto A.P., Delgado Rodrigues J. (2011). Consolidation of carbonate stones: Influence of treatment procedures on the strengthening action of consolidants. J. Cult. Herit..

[65] Sassoni E., Graziani G., Franzoni E. (2015). Repair of sugaring marble by ammonium phosphate: Comparison with ethyl silicate and ammonium oxalate and pilot application to historic artifact. Mater. Des..

[66] Khachani M., El Hamidi A., Halim M., Arsalane S. (2014). Non-isothermal kinetic and thermodynamic studies of the dehydroxylation process of synthetic calcium hydroxide Ca(OH)_2_. J. Mater. Environ. Sci..

[67] Delgado Rodrigues J., Grossi A. (2007). Indicators and ratings for the compatibility assessment of conservation actions. J. Cult. Herit..

[68] Stück H., Forgó L.Z., Rüdrich J., Siegesmund S., Török Á. (2008). The behaviour of consolidated volcanic tuffs: Weathering mechanisms under simulated laboratory conditions. Environ. Geol..

[69] Delgado Rodrigues J. (2001). Evaluación del comportamiento expansivo de las rocas y su interés en conservación/Swelling behaviour of stones and its interest in conservation. An appraisal. Mater. Constr..

[70] Jiménez-González I., Rodríguez-Navarro C., Scherer G.W. (2008). Role of clay minerals in the physicomechanical deterioration of sandstone. J. Geophys. Res. Earth Surf..

[71] Cherblanc F., Berthonneau J., Bromblet P., Huon V. (2016). Influence of water content on the mechanical behaviour of limestone: Role of the clay minerals content. Rock Mech. Rock Eng..

[72] Wheeler G. (2005). Alkoxysilanes and the Consolidation of Stone.

[73] Snethlage R., Ling H., Tao M., Wendler E., Sattler L., Simon S. (1996). The Sandstone of Dafosi—Investigation into Causes of Deterioration and Conservation Methods. ICOMOS Hefte Dtsch. Natl..

[74] Sassoni E., Graziani G., Franzoni E. (2015). An innovative phosphate-based consolidant for limestone. Part 1: Effectiveness and compatibility in comparison with ethyl silicate. Constr. Build. Mater..

[75] Mahy M., Van Eycken L., Oosterlinck A. (1994). Evaluation of Uniform Color Spaces Developed after the Adoption of CIELAB and CIELUV. Color Res. Appl..

[76] Favaro M., Mendichi R., Ossola F., Simon S., Tomasin P., Vigato P.A. (2007). Evaluation of polymers for conservation treatments of outdoor exposed stone monuments. Part II: Photo-oxidative and salt-induced weathering of acrylic-silicone mixtures. Polym. Degrad. Stab..

